# Systematic, Multimethod Assessment of Adaptations Across Four Diverse Health Systems Interventions

**DOI:** 10.3389/fpubh.2018.00102

**Published:** 2018-04-09

**Authors:** Borsika A. Rabin, Marina McCreight, Catherine Battaglia, Roman Ayele, Robert E. Burke, Paul L. Hess, Joseph W. Frank, Russell E. Glasgow

**Affiliations:** ^1^Denver-Seattle Center of Innovation for Veteran-Centered and Value-Driven Care (COIN), Denver VHA Medical Center, Denver, CO, United States; ^2^Department of Family Medicine and Public Health, School of Medicine, University of California San Diego, La Jolla, CA, United States; ^3^Adult and Child Consortium for Health Outcomes Research and Delivery Science, School of Medicine, University of Colorado, Aurora, CO, United States; ^4^Department of Family Medicine, School of Medicine, University of Colorado, Aurora, CO, United States; ^5^Department of Health System Management and Policy, Colorado School of Public Health, University of Colorado, Aurora, CO, United States; ^6^Department of Medicine, School of Medicine, University of Colorado, Aurora, CO, United States

**Keywords:** adaptation, RE-AIM framework, Stirman framework, mixed methods, pragmatic measures, assessment

## Abstract

**Background:**

Many health outcomes and implementation science studies have demonstrated the importance of tailoring evidence-based care interventions to local context to improve fit. By adapting to local culture, history, resources, characteristics, and priorities, interventions are more likely to lead to improved outcomes. However, it is unclear how best to adapt evidence-based programs and promising innovations. There are few guides or examples of how to best categorize or assess health-care adaptations, and even fewer that are brief and practical for use by non-researchers.

**Materials and methods:**

This study describes the importance and potential of assessing adaptations before, during, and after the implementation of health systems interventions. We present a promising multilevel and multimethod approach developed and being applied across four different health systems interventions. Finally, we discuss implications and opportunities for future research.

**Results:**

The four case studies are diverse in the conditions addressed, interventions, and implementation strategies. They include two nurse coordinator-based transition of care interventions, a data and training-driven multimodal pain management project, and a cardiovascular patient-reported outcomes project, all of which are using audit and feedback. We used the same modified adaptation framework to document changes made to the interventions and implementation strategies. To create the modified framework, we started with the adaptation and modification model developed by Stirman and colleagues and expanded it by adding concepts from the RE-AIM framework. Our assessments address the intuitive domains of *Who, How, When, What, and Why* to classify and organize adaptations. For each case study, we discuss how the modified framework was operationalized, the multiple methods used to collect data, results to date and approaches utilized for data analysis. These methods include a real-time tracking system and structured interviews at key times during the intervention. We provide descriptive data on the types and categories of adaptations made and discuss lessons learned.

**Conclusion:**

The multimethod approaches demonstrate utility across diverse health systems interventions. The modified adaptations model adequately captures adaptations across the various projects and content areas. We recommend systematic documentation of adaptations in future clinical and public health research and have made our assessment materials publicly available.

## Introduction

Implementing a program is like constructing a building. An architect draws upon general engineering principles (theory) to design a building that will serve the purposes for which it is designed…. However, the specific building that results is strongly influenced by parameters of the building site, such as the lot size, the nature of the site’s geological features, the composition of the soil, the incline of the surface, the stability and extremes of climate, zoning regulations, and cost of labor and materials. The architect must combine architectural principles with site parameters to design a specific building for a specific purpose on a specific site… This dynamic is mirrored in the rough-and-tumble world of the human services. Despite excellent plans and experience, ongoing redesign and adjustment may be necessary. [Bauman et al., (1)]

Health systems interventions are rarely ever implemented in precisely the same way across diverse, real-world settings. Changes to the original intervention and/or implementation protocol during the course of a program are described as adaptations in the dissemination and implementation literature and are receiving growing attention from researchers and practitioners alike (2, 3). By considering local culture, history, resources, characteristics, and priorities, interventions are more likely to lead to improved outcomes (2–5). Understanding the nature, origin, timing, and impact of these adaptations is crucial for many reasons. Adaptation information can provide contextual and process data and support the interpretation of study findings. It can also help identify which components of the intervention and implementation strategies worked and which components need to be modified in a given setting and for a given population, and can ultimately help answer the question of what components of an intervention work for what population, for producing what outcomes, under what circumstances. The information can then guide real-time or end-of-project improvements and refinements to intervention and implementation strategies and provides guidance for future scale up and scale out (6).

A critical piece in identifying adaptations to an intervention and implementation protocol is to find strategies to systematically evaluate and document the adaptations. The ideal pragmatic approach to documenting and evaluating adaptations happens in real time and throughout the lifetime of the project, is replicable, is unobtrusive to the users and beneficiaries of the intervention, has low complexity, is low cost and requires modest resources, provides both quantitative and qualitative information on the adaptation, assesses the adaptations from the perspective of multiple stakeholders, and uses multiple methods to generate rich data (7). Furthermore, an assessment strategy that can be applied across diverse settings, interventions, and implementation strategies would permit and encourage cross-study comparisons. Finding such an approach or combination of approaches poses a challenge, and there is little guidance in the literature to date.

Given the novelty of the field of adaptation research, there are numerous opportunities to develop and test methods to address questions such as which types of adaptations are most beneficial and which result in reduced fidelity and worse outcomes (2, 3). For example, are adaptations made before implementation any more or less helpful; are intentional adaptations more productive than unintentional ones; and are externally required (versus internally motivated) adaptations more disruptive?

In this study, we describe a mixed and multimethod approach to documenting and evaluating adaptations in the context of four, diverse, multisite health systems interventions and implementation efforts that are being applied in the Veterans Heath Administration (VHA) health-care system. We describe our adaptation documentation and evaluation strategies, including a modified framework and multiple methods used to collect data, provide preliminary findings on adaptations from four health systems intervention and implementation studies, and share lessons learned and possible applications of our methodology. Our assessment methods below are in the public domain and are available upon request from the authors, and we encourage their use, evaluation, and improvement.

## Methods

Section “Methods” provides a description of our four interventions, implementation strategies, and their settings; the adaptation framework and coding system used to guide our documentation and evaluation activities, and the details of the documentation and evaluation approach used across the four case studies.

### Setting and the Four Interventions

The VHA is the largest integrated health care system in the United States, providing primary and specialty health services to nine million enrolled Veterans. The VHA plays a lead role in improving the quality of patient care and health services through multiple initiatives, and the Quality Enhancement Research Initiative (QUERI)^1^ has been a central component of the VHA’s commitment to improve health care for Veterans (8). The Triple Aim QUERI is 1 of 15 currently funded QUERI programs and focuses on leveraging health-care data to identify actionable gaps in care, and to implement innovative health-care delivery interventions to improve the Triple Aims of VHA health care which are patient-centered care, population health, and value. The Triple Aim QUERI uses three projects to assess the feasibility and effectiveness of various interventions and implementation strategies unified by shared implementation models, measures, and approaches. In addition to these three projects, this manuscript includes a project from a sister VHA initiative funded through the VHA Office of Rural Health.

The four projects are described with their key characteristics in Table 1. As shown in Table 1, the four projects are diverse in the program focus area, clinical problem they address, target population, and the intervention format and delivery. The first project, titled Implementation of Extensible Methods to Capture, Report, and Improve Patient Health Status (Patient Reported Health Status Assessment), aims to utilize and implement the interactive voice response (IVR) to capture the pre- and postprocedural patient-reported health status for patients receiving elective catheterization laboratory procedures with intent to inform clinical care (9). The second project, titled Leveraging Data to Improve Multimodal Pain Care Through Targeted Telementoring (Multimodal Pain), aims to address barrier and facilitators to multimodal pain care in the VHA and to design and implement an intervention based on identified best practices to support primary care providers (10). The third project, titled Improving Veterans Transition Back to VA Primary Care Following Non-VHA Hospitalization (Community Transitions), focuses on care coordination of those Veterans admitted to non-VHA community hospitals for inpatient care and transition back to VHA primary care in a safe, patient-centered and timely manner (11). The fourth project, the Transitions Nurse Program (Rural Transitions), is a proactive, personalized, nurse-led and Veteran-centered intervention to improve access for rural Veterans to follow-up with their PACT teams following hospitalization at a larger urban VHA Medical Center (VAMC) (12).

**Table 1:** Characteristics of four health services intervention and implementation study and adaptation-related features.

Project name	Patient Reported Health Status Assessment	Multimodal Pain	Community Transitions	Rural Transitions
Problem addressed	Lack of standardized reporting of patient health status in setting of cardiovascular procedure	Delivering multimodal pain care through telementoring	Transitional care from non-network hospital to network primary care	Care coordination for rural Veterans during and post-discharge from a tertiary VHA Medical Center (VAMC) back to their Patient Aligned Care Team

Setting	VAMC	VAMC, community-based outpatient clinics	VAMC, community-based outpatient clinics, community hospitals	VAMC, community-based outpatient clinics

Population	Veterans, providers	Veterans, providers, staff	Veterans, providers, staff	Veterans, providers, staff

Intervention	To collect patient-reported health status information before and after percutaneous coronary intervention *via* an interactive voice response system and to integrate use of the health status data into routine clinical care	Leveraging data to identify gaps in the use of multimodal pain care and to train providers on best practices through telementoring	Integrated non-network hospital discharge care coordination program which includes nurse care coordination and health system changes including dedicated phone and fax lines for non-network hospitals and Veteran care identification cards	A transitions nurse at the VAMC who prepares patient for discharge and obtains a follow-up appointment, communicates with the Patient Aligned Care Team site about the discharge care coordination, follows up with the patient within 48 h after discharge, and engages with the rural Primary Care Provider and Registered Nurse to ensure continuity of care and information exchange

Implementation strategies	Audit and feedback; facilitation	Audit and feedback; facilitation	Audit and feedback; facilitation	Audit and feedback; internal and external facilitation; modified rapid process improvement workshop

Adaptation tracking methods and timeline	Real-time adaptations tracking form—ongoing; adaptations interviews with implementation team planned at two time points in the project, shortly after the roll-out and at the end of outcomes data collection	Real-time adaptations tracking form—ongoing; adaptations interviews with implementation team planned at two time points in the projects, shortly after the roll-out and at the end of outcomes data collection	Real-time adaptations tracking form—ongoing; adaptations interviews with implementation team planned at two time points in the project, shortly after the roll-out and at the end of outcomes data collection; direct observations planned as the intervention is expanded to additional sites	Real-time adaptations tracking form—ongoing; adaptations tracking database—ongoing; adaptation interviews with the implementation team at two time points in the project, shortly after the roll-out and at the end of outcomes data collection; direct observations planned at pre-implementation and post-intervention roll-out

Adaptation examples	Triggering data collection using site-specific electronic flags in the electronic health record	Changing facility eligibility criteria	Changing patient follow-up phone call script to make sure it communicates what we want for increased interest and enrollment in our program	Changing eligibility criteria

These four projects involved diverse groups of local, regional, and national operational partners from the inception of the projects. As part of this effort, each project actively engaged key operational partners and identified outcomes of direct relevance to these partners. The Multimodal Pain project partnered with the Office of Specialty Care and National Program for Pain Management; the Patient Reported Health Assessment project teamed with National Cardiology Program, Clinical Assessment Reporting and Tracking Program, Office of Analytics and Business Intelligence, Office of Quality, Safety and Value; the Community Transition project teamed with VHA Office of Community Care and VISN 19 Rural Health Resource Center-Western Region; and the Rural Transition project partnered with the Office of Rural Health and the Office of Nursing Services. Furthermore, each program utilized the Denver VHA Veteran Research Engagement Board. The Engagement Board brings Veterans and other health-care system stakeholders together to contribute to research in meaningful ways.

We involved Veterans at multiple phases of the project, including the design, implementation, adaptation, and evaluation. Individual projects have the opportunity to speak to Veterans from diverse socioeconomic and service backgrounds and receive rapid feedback and questioning to ensure the program being implemented has positive impact on Veterans, providers, and their care givers.

We also involved local VHA and non-VHA stakeholders where we learned about barriers and facilitators to current processes at the VHA and obtained suggestions for improvement. For example, the Community Transitions project teams conducted in-depth, pre-implementation assessment of the current process with VHA and non-VHA clinicians and staff as well as Veterans to understand the current transition of care process. Following this assessment, an intervention was designed to address barriers identified by these VHA and non-VHA participants. During the implementation phase, project team members reached out to VHA and community stakeholders to describe the intervention, its value to those involved and answer questions. During these meetings, project sub-teams were asked to tweak certain elements of the intervention that they then brought back to the larger team to discuss feasibility, value added and if it would improve health outcomes for Veterans. This iterative process continues as the intervention is ongoing and new community stakeholders are engaged. To keep adaptation information organized, each interaction is documented including the source of information, date of suggested change or improvement and comments.

This study was not considered research per VHA ORO policy 1058.05, therefore ethical review and approval was not required in accordance with the local legislation and institutional guidelines.

### Adaptation Framework

A number of adaptation frameworks currently exist. Many of them originated from the cultural adaptations literature that first acknowledged that interventions needed to be appropriately adapted to fit local cultural needs to be successful (2, 5). A systematic effort was conducted by Stirman and colleagues to identify the core characteristics of adaptations and modifications in the dissemination and implementation literature and resulted in the coding guide we reference as the Stirman adaptation and modification framework (4). The original Stirman framework provides a method to systematically code adaptations made to the content of the intervention (nature and level) and to the context in which the intervention is delivered as well as to document by whom the adaptations were made (4).

Hall and colleagues affiliated with our research group investigated adaptations in the primary care setting and found that to fully capture the nature and impact of adaptations in those applied settings it was necessary to expand the Stirman et al framework (13). They found the original Stirman framework categories useful, but further expanded the framework by adding constructs informed by the Reach, Effectiveness, Adoption, Implementation, and Maintenance (RE-AIM) framework^2^ to include why and when the adaptations were made and what the impact of the adaptations were (13, 14). The core constructs of the modified adaptation framework are described in Table 2. For ease of use and understanding by clinical and community leaders and staff who were interviewed, these domains were framed using intuitive categories of *Who, How, When, What*, and *Why* to classify and organize adaptations. For each area, coding categories are identified and listed in the table. This framework and coding system is used to inform the documentation and evaluation approach described in the next section.

**Table 2:** The Triple Aim Quality Enhancement Research Initiative Adapted Stirman Adaptation framework and coding system and interview questions.

Constructs	Coding categories	Interview question example
WHAT is modified?	Content (modifications made to content itself, or that impact how aspects of the treatment are delivered)Context (modifications made to the way the overall treatment is delivered)Training and evaluation (modifications made to the way that staff are trained in or how the intervention is evaluated)	WHAT Part 1: WHAT component or part of the intervention was changed in this adaptation; in other words, what was the nature of the change? For instance, was it a change to program content, format, delivery mode, staff delivering it, patients eligible, where, when or how it was delivered, or what?

At what LEVEL OF DELIVERY (for whom/what are modifications made?)	– Individual patient level – Group level – Individual practitioner level – Clinic/unit level – Hospital level – Network level – System Level	Implied from other questions

Context modifications are made to which of the following?	– Format – Setting – Personnel – Population	Was the change to program, content, format, delivery mode, staff delivering it, patients eligible, where, when, or how it was delivered or what?

What is the nature of the content modification?	– Tailoring/tweaking/refining – Adding elements – Removing/skipping elements – Shortening/condensing (pacing/timing) – Lengthening/extending (pacing/timing) – Substituting – Reordering of intervention modules or segments – Integrating the intervention into another framework (e.g., selecting elements) – Integrating another treatment into EBP (not using the whole protocol and integrating other techniques into a general EBP approach) – Repeating elements or modules – Loosening structure – Departing from the intervention (“drift”)	WHAT Part 2: How would you describe the type of change involved in this adaptation? Specifically, what did the change involve? Was something added, deleted, changed to better fit the patients, delivered at a different time or in a different way?

^a^WHEN: When during the project the adaptation was made?	– During planning stages, before intervention began – Early, during first few weeks of intervention – During the middle stages – At or close to the end of project	WHEN during the ____ program was this adaptation first made?

^a^HOW: How or on what BASIS was this change made?	– Based on our vision or values – Based on a framework (for example, PCMH) – Based on our knowledge or experience of working with patients – Based on QI data, summary information or results – Based on pragmatic/practical considerations (for example, “this is the only way it would work”) – Based on financial incentives/payment – Based on feedback or suggestions (Practice Facilitator/coach or other) – Other	HOW or on what BASIS was this change made—based on challenges implementing, on time concerns, on results or data you collected, on external or administrative concerns, feedback from patients or staff, or what basis?

^a^WHY: What was the purpose of the adaptation?	– Increase reach, participation, access – Increase effectiveness – Increase adoption by more clinics/settings or make intervention more aligned with organizational goals – Increase implementation/ability of staff to deliver intervention successfully	WHY Part 1: WHY was this adaptation made? For example, to get more people to participate, to make the program attractive to more settings, to increase its effectiveness, to make it easier to deliver, to make it easier to maintain or reduce costs, etc.? WHY Part 2: Was this adaptation a result of EXTERNAL factors (for example, change in organizational policies, reimbursement changes) or INTERNAL issues, such as workflow, changes in staff or similar issues?

BY WHOM are modifications made?	– Individual practitioner/facilitator – Team – Non-program staff – Administration – Program developer/purveyor – Researcher – Coalition of – stakeholders – Unknown/unspecified	WHO was responsible for first suggesting or initiating this change? Was this the person or persons the ones who implemented the change? (If not, who implemented the adaptation?)

^a^IMPACT: What are (subjective) short-term results of the adaptation?	– Are they positive, negative, no real impact? – Did the changes impact: ○ Reach/participation/access ○ Effectiveness ○ Adoption ○ Implementation/ability of staff to deliver intervention successfully ○ Maintenance	What was the short-term IMPACT of this adaptation? Did it have highly visible results? (For example did it result in more or less participation by patients, get more or fewer settings or staff involved, improve or decrease consistency of delivery, improve or reduce outcomes, reduce or increase time or costs? We understand that you may not have concrete outcomes results at this time—please tell us your best perception of the impact of this adaptation thus far.)

*^a^Additional constructs to original Stirman Adaptation Framework*.

### Documentation and Evaluation Approach

Our documentation and evaluation approach has two main components. We first created a robust documentation tool allowing for the real-time, ongoing tracking of adaptations throughout the course of the project, and we also used a semi-structured, multilevel, and multistakeholder interviews implemented at multiple time points. The combination of these two approaches is intended to provide rich data on adaptations to the intervention and implementation strategies, and inform the subsequent expansion of the intervention to additional sites in the VHA. Each of these approaches is described below in more detail. Lessons learned from the implementation of these approaches to date are summarized in Section “Results.”

#### Real-Time and Ongoing Tracking of Adaptations

The adapted Stirman framework and coding system was used to create a pragmatic, easy-to-use tabular worksheet to track adaptations as they occurred throughout the lifetime of the project. The original worksheet was pilot tested and refined to improve usability and decrease burden and obtrusiveness. The current version of the worksheet is used by project research personnel (i.e., project manager or coordinator) and is presented in Table 3 along with two examples of recorded adaptations. The real-time tracking sheet is designed to be used from the early planning stages of the project and is populated on a regular basis in consultation with frontline implementers. The goal of this assessment method is to allow for comprehensive capturing of changes made to the project and to improve recall during adaptation interviews described below.

**Table 3:** Real-time tracking of adaptations form and two examples.

Date of the modification	4/15/2016	6/2/2016
Description of the specific modification	ISurvey questions reordered—moved the Rose Dyspnea questionnaire to the end	Revised patient letter to include information about automated pre-procedural phone calls
Reason for the modification	To improve fluidity of the survey and enhance data capture	To prepare patients for data collection
BY WHOM are modifications made?	Researcher	Researcher
WHAT is modified?	Order of data collection	Content of the intervention
At what LEVEL OF DELIVERY?	Individual patient level	Individual patient level
CONTEXT modifications are made to…	Intervention format	Intervention format
What is the NATURE of the Content modification?	Tailoring/tweaking/refining	Tailoring/tweaking/refining
WHEN: When during the project the adaptation was made	During planning stages before began intervention	During planning stages before began intervention
WHY: What is the purpose of the adaptation?	Increase effectiveness	Increase implementation/ability of staff to deliver intervention successfully
IMPACT—What are (subjective) short-term results of adaptation?	Positive: impact effectiveness	Positive: impact implementation/ability of staff to deliver intervention successfully

The implementation teams used different strategies to support the implementation of the real-time tracking form across our four projects. These strategies included first, the addition of a standing agenda item to weekly/biweekly meetings with implementers to ask about challenges they encountered during the implementation of the project and whether they needed to make or planning on making any changes to address these challenges; the discussion and adaptations data collection was facilitated by both implementation and clinical team leads. Second, some projects converted their regular team meeting documents (such as action items and minutes) into data that fit into the main constructs/coding areas from the adapted Stirman Framework to facilitate the documentation of relevant information related to changes in the project. Third, in some projects, the worksheet was embedded in the tracking database to be completed by the frontline implementers (e.g., Rural Transitions nurses in the participating sites) with guidance from the research team to track adaptations in real time. Fourth, in one of the projects notes made from periodic direct observations of intervention delivery were used to clarify, add to, or enhance adaptation descriptions; these included the field notes and process maps from site visits. In this project, a team consisting of an implementation specialist and a research nurse conducted site visits to all expansion sites approximately 6 months after intervention initiation to directly observe the delivery of the intervention and document adaptations made since program roll-out at each site. The observational data are used to construct intervention process maps and provide additional contextual factors for the implementation evaluation. The remaining projects are planning to adopt this approach when the interventions are expanded to additional sites. Information from the real-time tracking system is used to create a list of adaptations as well as to support interviews (i.e., help with recall).

#### Semi-Structured, Multilevel, and Multistakeholder Interviews

A semi-structured interview guide and coding system adapted from that used by Hall and colleagues (13) tailored to the context of our four projects was drafted and pilot tested. Example questions and probes from the interview guide as they align with the various construct/coding categories are listed in Table 2. First, interviewees are asked to identify all changes they made to the original intervention or implementation strategy protocol. Then, they are asked to identify the most important changes made to the intervention or implementation strategy and to list them in the order of perceived importance, with the first change being most important. Detailed follow-up questions are then asked related to the change that was deemed most important by the interviewee; if time permits, follow-up questions are asked about the additional changes mentioned in the beginning of the interview. In some cases, adaptations documented in the real-time tracking document were systematically used to improve interviewee recall and remind interviewees about important changes that happened during the implementation of the intervention. The semi-structured adaptation interviews are designed to be conducted at two time points or more during the project, including soon after implementation of the intervention (within 3–6 months) and at the end of the project. The full interview is available at https://goo.gl/PDGWtf.

Each project identifies a set of stakeholders to interview including frontline implementers and research personnel. Interviews are audio recorded, transcribed, and coded. The qualitative content is managed using Atlas ti. software package. The qualitative analytical team uses consensus-building to discuss the emergent codes and themes and to resolve differences in coding. Data are summarized in the form of adaptation lists. Each project plans to conduct two waves of interviews, one soon after implementation and another right at the end of the project. We are planning on interviewing up to 10 people in various roles in the implementation process for each wave and project. Findings from the earlier wave of the interviews will be used to inform refinements to our interventions and implementation strategies and approaches for subsequent expansion of the interventions as well as to support interpretation of our findings at the end of the project. We will also use information from these interviews (in combination with the data emerging from the real-time tracking system) to create an adaptation guide for future implementers.

## Results

In this section, we share preliminary results and lessons learned from our four projects. All four of these projects are in progress and at various stages of the planning and implementation continuum.

### Real-Time and Ongoing Tracking of Adaptations

The real-time tracking system has been implemented across all four projects. We have documented a total of 46 adaptations to date across the four projects (average of 12 per project, most of which occurred shortly after initiation of the intervention). Table 3 lists two specific examples and demonstrates what the real-time tracking document look like in action. Most adaptations documented to date have been related to the intervention delivery, such as defining and fine-tuning enrollment criteria in the Rural Transitions project, initiation of the IVR calls in the Patient Reported Health Assessment project, and recruitment materials in the Community Transitions project.

The real-time tracking sheet is used by project managers or coordinators on a weekly basis. It requires approximately 3–5 min to complete the tracking sheet for each adaptation. Key adaptations documented here included scope of the intervention, its delivery and evaluation plans for the Community Transitions project, expansion of the enrollment criteria in the Rural Transitions project; modifications to the IVR calls delivery in Patient Reported Health Assessment project. Some key lessons learned from the use of the tracking sheet are summarized in Table 4. Positive feedback included the perceived usefulness of documenting information in a structured manner which allows for ready retrieval at a later time, and help with identifying core components of the intervention and implementation protocol. Some of the lessons include strategies on how to implement the real-time tracking system [e.g., the need to set reminders (calendar reminder)], the importance of checking in regularly with the project team along with using the tracking sheet, and the need to communicate with frontline implementers about possible changes/adaptations as the research team is not always aware all changes made by frontline staff. Another challenge that was identified was that results of adaptations may not be clear until weeks or months after the change, making it difficult to record this information.

**Table 4:** Lessons learned from using the real-time tracking system and interviews.

Project	Project role	Tracking tool used	Reflections/feedback/lessons learned
Community Transitions	Project coordinator	Real-time tracking form	– Easy to forget real-time tracking, recurring calendar reminders are useful – Helps when need to go back and look at certain decisions made with the team – Easy to forget if there are a lot of changes happening to the intervention in a short period of time

Patient Reported Health Status Assessment	Project coordinator	Real-time tracking form	– Helps clarify core intervention and implementation components – Critical to talk to line implementers—decision-maker and research team may be unaware of adaptations – Helps document exactly what component was adapted—when, why, how, and by whom (Stirman framework as a guide)

Multimodal Pain	Project coordinator	Real-time tracking form	– Helps keep audit trails up to date so time is not wasted digging through emails/notes – Need to organize inbox folders by project and keep emails related to the project – Need to set reminders in outlook (or other system) to update tracking sheets – Important to document everything even if it does not seem important at the time

Rural Transitions	Implementation specialist	Real-time tracking form	– Challenging to track in real-time simultaneously at multiple sites and based on feedback from multiple team members

Rural Transitions	Research Transitions coordinator, RN	Real-time tracking form	– Easy to check in with teams as it became a standing part of the agenda – New way to track changes in the project—was not familiar and as convenient – Useful system and provided valuable information to refer back to

Rural Transitions	Qualitative analyst 3	Real-time database tracking	– Need to remind the site implementation teams to track in the database

Rural Transitions	Qualitative analyst 1	Adaptation interview guide	– Introduction to the interview was too long – Difficult to have interviewees first list all adaptations and then go through the questions for each adaptation. Would be more organic to have them mention the first adaptation, then ask follow-up questions, then probe for more adaptations – Some of the probing questions felt repetitive – Might be helpful to do interviews in the first couple months of implementation since that is when most of the adaptations seemed to take place

Rural Transitions	Qualitative analyst 2	Adaptation interview guide	– Introduction was too long – Participant did not feel they had made adaptations, so some of the follow-up questions were awkward – Difficult to fill in table while conducting interview, need to code after interview is complete

Rural Transitions	Qualitative analyst 3	Adaptation interview guide	– Interview questions needed additional clarifications, so the follow-up questions and probes were helpful to clarify – Some questions seemed repetitive (e.g., How and Why) – Many times, an interviewee would start describing the details of the adaptations and answer all the follow-up questions without prompting

### Semi-Structured, Multilevel, and Multistakeholder Interviews

Adaptation interviews have started in three of the four projects (Rural Transitions, Community Transitions, and Patient Reported Health Assessment). We conducted 11 interviews with site implementers (transitions nurses and champions) in Rural Transitions, three interviews with program staff and transitions nurse in Community Transitions; and one interview with implementers in the Patient Reported Health Assessment project. Interviews last an average of 45 min. Table 4 summarizes early lessons from our adaptation interviews. Key reflections include the realization that some interview questions might not be different enough to produce distinct responses (e.g., questions about WHY and HOW), the sequence of the interview was not always optimal (e.g., would prefer to ask details about adaptation when first mentioned instead of waiting to list all adaptations), probes were helpful in most cases, the introduction for the interview was too lengthy, and it can be challenging to record information about the adaptation in the interview table while conducting the interview. One unexpected finding from these early interviews was that an adaptation of the Rural Transitions intervention was to limit the number of eligible patients to enroll to avoid the burnout of the transitions nurse. Information from these early interviews has been used to inform the intervention roll-out process for the subsequent expansion of the Rural Transitions program by providing guidance on the enrollment strategies for the on-coming sites. Finally, the timing for conducting the early wave of interviews were somewhat delayed by the competing demands of the implementation of the intervention.

## Discussion

Our adaptations project has conceptualized assessment methods, developed and adapted multimethod procedures, and is applying them across four diverse projects and content areas. The methods appear to be feasible, informative, and applicable across different clinical targets, interventions, research projects, and settings. As discussed below, preliminary results appear to be promising and investigations are ongoing. Our focus throughout, in accord with implementation and dissemination principles (15, 16), has been on multiple methods, multiple contextual levels, and rapid, pragmatic assessment strategies (17). We summarize overall experiences to date, lessons learned, strengths and limitations of the developed approaches, and opportunities and needs for future research.

Our methods are purposively designed to be broadly applicable but require some training and dedicated time for non-researchers to utilize. In addition, these methods have low to moderate burden, produce rapid results, and are flexible to fit different content areas. None of our assessment methods require large amounts of time or high levels of expertise. These are important features of pragmatic assessment, which has recently received increased attention in implementation science (7, 18–23). Importantly, busy clinical staff are not asked to complete lengthy questionnaires or spend lots of time in added meetings or assessment procedures. The most time-consuming activities including tracking records, conducting and analyzing interviews, and conducting observations can be completed by project managers or research assistants without high levels of advanced education. Many activities, especially the tracking documentation, can be accomplished by keeping good records during existing project management and supervision activities.

Our assessment methods are flexible and can be tailored to different projects and purposes. They can be adapted to a particular project in terms of the sources and levels of information collected (e.g., CEOs and macro-level adoption decisions; providers and guidelines application; front line delivery staff and implementation actions). Tracking and observational data can be collected in the context of any combination of team meetings, site visits, other assessment procedures, quality control contacts, direct observations, phone call check-ins or other opportunities. These rapid and frequent assessment methods can be used iteratively to inform future inquiries and adaptations. Thus far, we have made use of this feature by tracking data to be assessed in more detail in structured adaptation interviews (13).

An optional feature of our assessment methods can be viewed as either a strength or limitation. On the one hand, the procedures, coding categories, areas of focus, and results assessed can vary over time and are informed by accumulating data. From a traditional efficacy research and psychometric perspective, some of these updates and assessment modifications may be seen as methodologically problematic. From this perspective, assessment should be defined before data collection and applied in a standardized fashion regardless of results (and results may not be reviewed until project conclusion). We understand this perspective, and note that flexible and iterative use of our methods is optional and not required if these features are not desired. On the other hand, in the spirit of rapid use of research results and improvement science (24), actionable information can and should generally be useful to inform intervention and implementation adaptations, which are likely to occur in any case, but otherwise be less informed by data (3).

This study and our methods raise two important additional questions (1) why, how, what types of adaptations are successful (or not) and (2) how might one “optimize” adaptation of an intervention at the design stage for maximum success. We do not yet have data on the first issue but will at the conclusion of the four projects. We do collect “immediate perceived impact” of the staff and interviewee on the forms, but these are subjective and do not address delayed effects. The second issue of the use of these adaptation data on how to optimize intervention—and implementation strategies—is clearly important and extremely complex (25). Some researchers do not feel it is appropriate to modify an intervention following development of an initial protocol, and others have proposed both adaptive or SMART designs and use of modeling approaches to address these issues (26). More detailed discussion of these issues is provided, for example, in Riley and Rivera (27).

Our experiences to date have also revealed challenges and limitations to these assessment procedures. First, optimal use of our multiple methods requires in-depth knowledge of project intervention and implementation strategies. Sometimes assessment staff are not in contact with intervention planners or implementers and are not informed about procedural details. Our methods can still be used in such situations, but will likely not be as specifically useful to those projects in terms of informing future directions. Our team had initial difficulties in differentiating adaptations made to intervention components versus implementation strategies (such as audit and feedback or facilitation) (28), partially because the grant project applications funding our assessments were not always clear on these distinctions. Our methods can be used to assess adaptation to either or both intervention components or implementation strategies. In some cases, these distinctions may be important for either scientific or application purposes; in other cases, they may not. Furthermore, our current project only allowed for the administration of the interview portion of our methodology at two time points. An additional interview during the planning/pre-implementation phase would be ideal but not essential.

Since the projects involved had moderately specific protocols concerning intervention components, and especially implementation strategies (rather than being scripted and manualized interventions), it was sometime challenging to understand precisely what the intervention component was and whether it was adapted or implemented as originally intended. Other limitations include that thus far we have not conducted formal reliability or validity assessments.

Our adaptation assessment methods are based upon the Sitrman and RE-AIM frameworks, both of which have been used in multiple settings and found valid and useful (4, 13, 29, 30). However, the *specific* assessment instruments used in this study, while demonstrating high face validity, have not been subjected to formal psychometric testing. Since these are new measures, the analytic implications of these methods are unclear. Many potentially useful variables (e.g., timing, source, content, and purpose of adaptation) can be coded from these methods, but it is not clear which are most important, their interrelationships, or exactly how they should be analyzed (e.g., continuous versus dichotomous variables).

Furthermore, it does take time and effort to collect these adaptation data and their value needs to be weighed against alternative uses of resources. We have tried to minimize the time and burden on both staff (e.g., recoding tracking form data during regular meetings) and delivery staff (doing only two interviews at convenient times), but these activities might not be high enough priorities for some projects to justify the time.

Another important consideration is the way in which impact is tracked across time using the proposed approach. While we do not systematically follow-up on tracking data to evaluate the impact of adaptations (we do assess initial impact, but some adaptations are of course delayed in time), there are concurrent assessments of some separate process and intermediate outcome measures.

Finally, as is the case with multiple methods in general, it is not clear exactly how to integrate data from multiple sources (31–33).

Despite these limitations, we conclude that these multiple adaptation assessment methods are useful and worthy of further investigation. In addition to formal psychometric testing regarding reliability and concurrent validity, we especially recommend study of the extent to which these methods are useful for iteratively informing intervention and implementation modifications during a project. Future studies could also evaluate the value and cost-effectiveness of these brief, pragmatic assessment methods compared with more traditional evaluation procedures. Future research is indicated that helps inform the overarching question of which assessment methods are most useful in what settings.

## Ethics Statement

This study was not considered research per VHA ORO policy 1058.05.

## Author Contributions

BR conceptualized and prepared the first draft of the paper, developed the assessment methods and tool, led the interpretation of the data interpretation, and reviewed the final draft of the paper. MM participated in the conceptualization and development of the first draft of the paper, participated in the development of the assessment methods and tools, led the data collection and data analysis, participated in the interpretation of the data, and reviewed the final draft of the paper. CB participated in the conceptualization and drafted sections of the paper, participated in the development of the assessment methods and tools and the data interpretation and reviewed the final draft of the paper. RA participated in the development of the first draft of the paper, the development of the assessment methods and tools, data collection, data analysis, and interpretation of findings, and reviewed the final draft of the paper. RB participated in the drafting of sections of the paper, serves as the PI for the Rural Transition project, participated in the interpretation of the data and reviewed the final draft of the paper. PH participated in the drafting of sections of the paper, serves as the PI for the Patient Reported Health Assessment project, participated in the interpretation of the data and reviewed the final draft of the paper. JF participated in the drafting of sections of the paper, serves as the PI for the Multimodal Pain project, participated in the interpretation of the data and reviewed the final draft of the paper. RG co-led the conceptualization of the paper with BR, developed the first draft of the paper with BR, developed the original assessment tool, participated in the development of the revised assessment methods and tool, participated in the data interpretation, and reviewed the final draft of the paper.

## Conflict of Interest Statement

The authors declare that the research was conducted in the absence of any commercial or financial relationships that could be construed as a potential conflict of interest. The reviewer SY and the handling Editor declared their shared affiliation.

## Footnotes

^1^https://www.queri.research.va.gov/ (Accessed: March 10, 2018).

^2^www.re-aim.org (Accessed: March 10, 2018).
